# Performances of PTFE and PVDF membranes in achieving the discharge limit of mixed anodic oxidation coating wastewaters treated by membrane distillation

**DOI:** 10.1007/s11356-024-33830-9

**Published:** 2024-06-03

**Authors:** Oruc Kaan Turk, Ali Zoungrana, Mehmet Cakmakci

**Affiliations:** Department of Environmental Engineering, Yildiz Technical University, 1,Davutpasa Campus 34210 Esenler, Istanbul, Turkey

**Keywords:** Electroplating, Membrane distillation, Wastewater, Anodic oxidation, Sulfate, Chemical oxygen demand

## Abstract

The mixed wastewater generated by anodic oxidation coating facilities contains high levels of various contaminants, including iron, aluminum, conductivity, chemical oxygen demand (COD), and sulfate. In this study, the effectiveness of the membrane distillation (MD) process using polytetrafluoroethylene (PTFE) and polyvinylidene fluoride (PVDF) membranes was investigated to treat mixed wastewater from an anodized coating factory. The results indicate that both hydrophobic membranes effectively removed targeted contaminants. However, the PTFE membrane achieved higher removal efficiencies, with over 99% removal of sulfate, conductivity, iron, and aluminum, 85.7% of COD, and 86% of total organic carbon (TOC). In contrast, the PVDF membrane exhibited a significant decline in removal efficiency as the temperature increased and performed well only at lower feed temperatures. The PTFE membranes outperformed the PVDF membranes in treating chemically intensive anodic oxidation wastewaters. This superiority can be attributed to the PTFE membrane's morphology and structure, which are less influenced by feed water temperature and chemicals. Additionally, its slippery surface imparts anti-adhesion properties, effectively preventing membrane fouling, and maintaining the treated water quality and flux for longer operation time.

## Introduction

Distillation, a separation technology with a centuries-old history, has been widely employed in various industries and has found applications in water treatment and desalination. In response to the performance limitations and high energy consumption inherent in the distillation process, industries have been exploring higher-performance, cheaper, and environmentally friendly separation technologies to replace the traditional distillation method (Kiss and Kattan Readi 2018). In recent years, membrane processes, an advanced water treatment method, have experienced increasing applications in various areas, including domestic and industrial wastewater treatment, drinking water treatment, and desalination applications (Bera et al. 2022; Kamali et al. 2019; Kesari et al. 2021; Mojiri and Bashir 2022). With the significant ongoing advancements in material science and membrane technology, the membrane distillation process has evolved, integrating a distillation process and membrane separation (Ahmed et al. 2020; Alkhudhiri et al. 2012; Basile et al. 2015; Biniaz et al. 2019; Deshmukh et al. 2018; Drioli et al. 2015; Ghaffour et al. 2019).

Membrane distillation (MD) is an advanced membrane technology with main applications in seawater and brackish water desalination (Ahmed et al. 2020; Basile et al. 2015; Susanto 2011). On the other hand, the MD process has been widely applied for the purification of wastewater generated by a variety of industries, but also for the recovery of valuable materials (metals) and the reduction of harmful pollutants in wastewater prior to discharge (Aijaz et al. 2022; Biniaz et al. 2019; Bramsiepe et al. 2012; Foureaux et al. 2020; Kamali et al. 2019; Kiss and Kattan Readi 2018; de Sousa Silva et al. 2021; Zheng et al. 2015). The MD technique uses specially designed hydrophobic membranes that allow water vapor to pass through due to a thermally driven mechanism, all while maintaining their structural integrity (Souhaimi and Matsuura 2011; Tibi et al. 2020; P. Wang and Chung 2015). This method operates at lower temperature and pressure compared to traditional distillation and pressure-based membrane processes, while still providing excellent purification performance by effectively removing all non-volatile compounds, including organics and various ions such as sulfate, aluminum, and iron (Boubakri et al. 2015; Cath 2010; Hou et al. 2014; Kezia et al. 2015; Souhaimi and Matsuura 2011; Tomaszewska 2001; Van der Bruggen and Vandecasteele 2002; Zoungrana et al. 2017; Zoungrana et al. 2016). MD has been reported to be particularly effective for treating industrial wastewater, including mixed anodic oxidation wastewater, which often contains similar pollutants to those found in various industries such as electroplating, mining, clothing, battery manufacturing, and mineral processing (Helen Kalavathy and Miranda 2010; Molinari et al. 2008). Several patents and studies in the literature demonstrate the applicability of MD systems, either alone or in combination with other processes, for desulfurization and heavy metal removal (Attia et al. 2017; Haıyang et al. 2019; Hubadillah et al. 2017; Lou et al. 2020; Zhumei et al. 2016; Zoungrana et al. 2016; Zoungrana et al. 2016). While research specifically on treating wastewater from anodic oxidation coating facilities is limited, existing studies suggest membrane separation techniques, including MD, hold promise for effectively treating these wastewaters.

Aluminum is a widely used metal worldwide, and anodized coating, a unique electrochemical process, imparts diverse properties to aluminum. While various chemicals can be employed to achieve different properties during the coating process, sulfuric acid stands out as the most commonly used electrolyte in this particular coating method (Kaufman 2019; Runge and Pomis 2000; Sulka 2020). The anodic oxidation process generates two separate streams, acidic and alkaline. The wastewater intended for treatment is collected by mixing these streams, resulting in a neutral pH range. However, the mixed wastewater still contains pollutants such as iron, aluminum, high conductivity, COD, and sulfates at high concentrations (Vargel 2004). Conventional treatment of anodic oxidation wastewater often relies on chemicals like sodium hydroxide (NaOH) and calcium hydroxide (Ca(OH)_2_). These chemicals can be expensive and generate sludge, which is costly to manage. According to Mymrinet al., the annual global production of anodized aluminum amounts to 450,000 tons, with an equivalent amount of aluminum anodized sludge generated for each 1 kg of anodized aluminum manufactured. This production is distributed among various countries, with Japan accounting for 39%, the USA for 22%, the European Union for 22%, and the remaining 17% being produced by other nations. This contributes to approximately 100,000 tons per year of aluminum anodized sludge generation in the European Union (Mymrin et al. 2021). Advanced oxidation processes and biological degradation methods have been investigated and proven to be inappropriate for anodizing industry wastewater treatment. In fact, oxidative degradation is hindered by heavy metal non-biodegradability and the scavenging of •OH radicals by anions like phosphates, sulphates, and nitrates present in the anodic oxidation wastewaters. Biological degradation is also unsuitable due to the solution pH and high heavy metal concentrations, which are inhospitable for most microorganisms (Ighalo et al. 2022). Separation processes such as nanofiltration, reverse osmosis, and adsorption techniques have demonstrated effective pollutant removal when dealing with various pollutant species in anodic oxidation wastewaters (Ali et al. 2020), however, the nature and constitution of anodizing industry wastewater require membranes with unique properties to successfully achieve an acceptable discharge limit. The application of advanced treatment methods is crucial to achieving better discharge standards for these wastewaters and reducing their damage to the environment.

Membrane distillation is an advanced treatment technology that has been demonstrated to be a viable alternative technique for treating non-vapourable compound-polluted water. A previous study demonstrated that such a process can be effective in the treatment of acidic anodic oxidation wastewaters (Türk et al. 2022). Ali et al. achieved 99.4% conductivity removal and extracted high-purity sodium sulfate crystals from anodic oxidation wastewater using combined MD/Membrane Crystallization (MD/MCr) with polypropylene (PP) hollow fiber membranes (Ali et al. 2019). It is worth noting that various parameters control the performance of MD processes, among which the hydrophobic membrane properties, the characteristics of the water to be treated and the operation parameters are the most important. As hydrophobicity is a fundamental requirement in the membrane distillation process, the membrane material should possess intrinsic hydrophobic properties, or its surface must undergo modification to acquire hydrophobicity. Commonly used membrane materials for MD include PP, polytetrafluoroethylene (PTFE), and polyvinylidene fluoride (PVDF) (Khayet 2011). Despite the critical role of hydrophobic membranes in MD, limited research has investigated the suitability of commercially available membranes for treating chemically intensive wastewater, especially those containing challenging pollutants like anodic oxidation wastewater. Existing studies lack investigation into the specific chemicals and their threshold concentrations that membranes can resist. Additionally, the influence of membrane structure on resistance is not fully understood, as some membranes show resistance to certain pollutants while others do not. Recognizing that the polymers used in membrane fabrication significantly impact their properties, this study aims to evaluate the influence of the structural properties of commercially available PTFE and PVDF hydrophobic membranes on their performance and integrity when treating anodic oxidation wastewater in an MD process. All Abbreviations and symbols used in this paper will be defined and reported in the “Abbreviations/symbols” section.

## Materials and methods

### Characterization of wastewater from an anodization plant

To conduct the present study, wastewater samples were collected from an anodization plant located in Istanbul, Turkey, which produces both acidic and alkaline wastewater streams. A mixed wastewater sample, resulting in a neutral pH, was collected for the study. An overview of the wastewater characteristics is provided in Table 1. According to the Turkish Water Pollution Control Regulation, the maximum allowable levels for the discharge of treated wastewater effluent into the environment 3 mg/L Al, 100 mg/L COD, and pH in the range of 6 to 9. Discharge regulations of sulfate limit its content to 1700 mg/L to protect infrastructure systems (SKKY 2004).

**Table 1:** Characteristics of anodization plant wastewater

Parameters	pH	COD mg/L	TOC mg/L	Sulfate mg/L	Conductivity μS/cm	Aluminum mg/L	Iron mg/L
Wastewater	6.91	322	81.3	17238	17110	625.5	10.35

### Membrane distillation system and operation

**Fig. 1:**
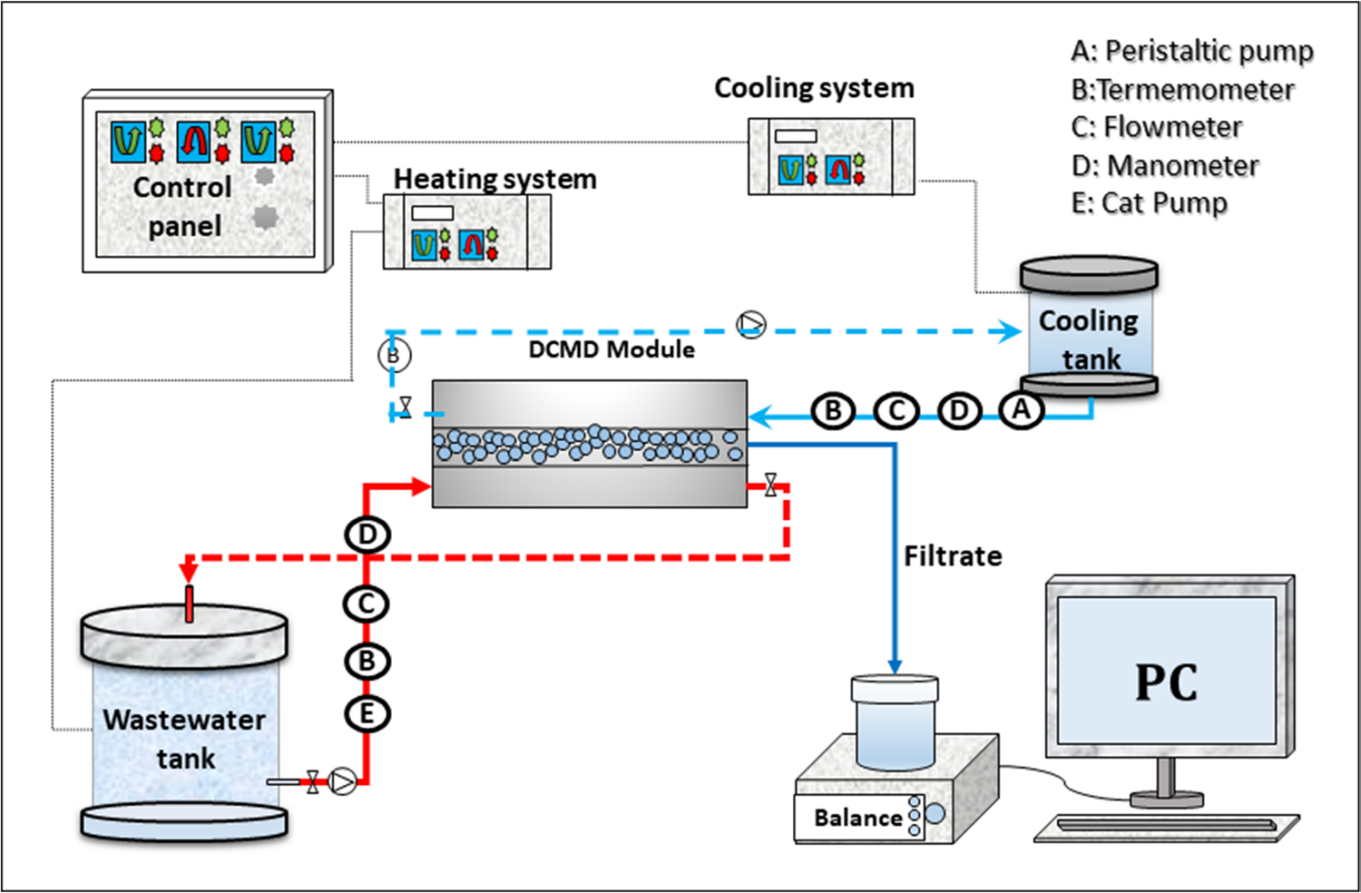
Schematic representation of the DCMD setup used in the study

A modified lab-scale flat-sheet direct contact membrane distillation (DCMD) system was used in the present study. The modification intended to reduce the temperature polarization and the heat loss by conduction suffered by the DCMD process (Zoungrana et al. 2016). An MD module with an effective membrane surface area of 0.015 m^2^ was used. The flow rate, temperatures, and operating pressures were continuously monitored using a flow meter, digital temperature probes, and manometers, respectively. Additionally, a digital balance linked to a personal computer was employed to measure the permeate mass and compute the flux. The temperature of the cooling water was kept constant at 10 °C, using a heat-exchanger. The wastewater was pumped into the MD module at a flowrate of 4.5 L.min^−1^, using a CAT pump 2SF35SEEL-stainless steel direct-drive plunger pump (Minneapolis, MN, USA) and operated for 2 h at four different heating temperatures: 50, 60, 70, and 80 °C. The schematic representation of the current MD process is given in Fig. 1.

### Hydrophobic membranes used

The MD system was operated using two distinct hydrophobic membranes produced from PTFE and PVDF polymers, respectively, with varying pore sizes, 0.45 μm of and 0.22 μm. All the membranes were manufactured by Membrane-Solution-LLC (Shanghai, China) and their physical properties are presented in Table 2. The membranes were conditioned at room temperature around 24 ± 1 °C before being used in the MD process.

**Table 2:** Properties of PTFE and PVDF hydrophobic membranes used in the MD process

Membrane material	Pore size (μm)	Thickness (μm)	Flow rate (mL/min/cm^2^ at 0.7 bar)	Bubble point (bar)	Liquid entry pressure (LEP) (bar)	Contact angles (°)
PTFE	0.22	210±30	12.75±1.75	1.3±0.2	4.75±0.75	140.88
PTFE	0.45	195±25	68.5±5.5	0.75±0.2	4.5±50	140.49
PVDF	0.22	160±20	5.4±0.9	1.25±0.15	3.75±0.75	100.32
PVDF	0.45	150±15	13.5±3	1.1 ±0.1	3.25±0.25	98.51

### Analysis and methods

The treatment performance of the MD process depends on two key factors: membrane rejection and permeate flux. Membrane rejection (R) represents the fraction of contaminants separated from the permeate stream and is typically expressed as a removal efficiency percentage (as shown in Eq. 1). Membrane permeate flux (J) quantifies the amount of water passing through a unit area of the membrane within a specified unit of time (as indicated in Eq 2).

1 $$R=\frac{\textrm{Cf}-\textrm{Cp}}{Cf}\ast 100\%$$

where R is the foulants removal efficiency (%), Cf and Cp are the concentration of the contaminant in the feed solution and in the permeate solution, respectively.

2 $$J=\frac{\textrm{Qp}}{A}$$

J is the membrane flux (L/h/m^2^), Q_P_ represents the permeate flow rate (L/h), and A stands for the surface area of the membrane (m^2^).

The wastewater quality analysis in this study was conducted following the guidelines outlined in the Standard Methods (SM) for the Examination of Water and Wastewater published by the American Public Health Association (APHA). Table 3 provides an overview of the water quality analyses and corresponding methods utilized in this study. Additionally, the morphology and structure of both the neat and used membranes were analyzed using contact angle measurements (Attension, Theta Lite Optical Tensiometer), scanning electron microscopy (SEM), and Fourier Transform Infrared Spectroscopy (FTIR) (Agilent Technologies, Cary 630 FTIR Spectrometer).

**Table 3:** Water quality analyzes and methods

Parameter	Analysis
Conductivity	Thermo Scientific Orion 5-Star Multimeter
pH	Thermo Scientific Orion 5-Star Multimeter
Temperature	Thermo Scientific Orion 5-Star Multimeter
COD	SM-5220 D
TOC	SM-5310 B
Aluminum	SM-3120 B
Iron	SM-3120 B
Sulfate	SM-4500-SO_4_^2-^ E

## Results and discussion

### Performance optimization studies with MD

MD is a 3rd generation treatment technology used for the effective removal of ions, particles, and non-volatile materials in water and wastewater. In the present study, the MD was operated for 2 h at different feed temperatures using PVDF and PTFE membranes with both pore sizes of 0.45 μm and 0.22 μm, to study a comparative performance of each membrane for the treatment and purification of anodic oxidation wastewater. The treated wastewater quality, the membrane flux, and the membrane resistance to wetting were the main parameters of the investigation.

The results displayed in Table 4 indicate that the contaminants’ removal efficiencies are affected by the membrane pore size and increasing feedwater temperature. An increase in both the feed water temperature and the membrane pore induced a decrease in the contaminants removal efficiency. On the other hand, a particular increase in the organic matter concentration was observed in the MD filtrate, which is believed to result from the evaporation of some readily evaporable organic matters in the feed solution. Although increasing feed water temperature affected the foulant removal efficiencies of both PVDF and PTFE membranes, a significant decrease in the foulant removal efficiency of PVDF membranes was observed as the feed water temperature increased to 70 °C and above. It is plausible that the diminished retention ability observed in the study was partly attributed to the contact angles of the clean membranes. Specifically, the contact angles that are approximately 90 degrees may have led to membrane wetting upon exposure to higher feed water temperatures, consequently resulting in a weakened retention capacity. This phenomenon could potentially explain the observed outcomes, indicating the critical role of membrane properties and their interactions with the feed water in the performance of the MD system. Another reason is that as the temperature rises, evaporation becomes more efficient and as more vapor passes through the membrane, ion transport is thought to become more important. PTFE membrane filtration under various operating conditions and pore sizes demonstrated a significant reduction in sulfate concentration in the permeate solution. The permeate’s sulfate content was much lower than both the World Health Organization’s (250 mg/L) and the Istanbul Water and Sewerage Administration’s (1700 mg/L) discharge regulations. This demonstrates the potential of PTFE membrane filtration for sulfate removal from water sources, providing a promising solution for sulfate pollution in industrial and domestic wastewater treatment. On the other hand, PVDF membranes were not able to meet the limit values for sulfate concentration in the permeate solution at any of the utilized pore sizes and operating conditions. However, it maintained effective heavy metal removals from the wastewater. Prior MD studies involving various wastewaters have demonstrated a superior performance of PTFE over PVDF membranes, which is attributed to PTFE enhanced operability and higher mass transfer coefficient (J. Zhang et al. 2010).

**Table 4:** Pollutant concentrations and removal efficiency in permeate after MD treatment

Membrane type and pore size	Feed temperature	Conductivity		Sulfate		COD		TOC		Aluminum		Iron	
PTFE 0.22 μm	°C	μS/cm	%	mg/L	%	mg/L	%	mg/L	%	μg/L	%	μg/L	%
	50	36.8	99.8	40.2	99.8	<20	>94	0.4	99.5	0.28	>99.9	0.23	>99.9
	60	78.4	99.8	48.7	99.8	<20	>94	2.7	96.7	0.31	>99.9	0.23	>99.9
	70	98.3	99.5	86.3	99.5	<20	>94	6.5	92	0.32	>99.9	0.25	>99.9
	80	124.2	99.5	97.6	99.5	<20	>94	7.3	91	0.48	>99.9	0.41	>99.9
PTFE 0.45 μm	50	41.2	99.8	40.6	99.8	<20	>94	5.4	93.4	0.35	>99.9	0.35	>99.9
	60	101.6	99.4	51.5	99.7	<20	>94	8.9	89.1	0.48	>99.9	0.48	>99.9
	70	150.3	99.2	92.4	99.5	22	93.2	8.6	89.4	0.58	>99.9	0.58	>99.9
	80	178	99	110.2	99.4	36	85.7	11.3	86.1	0.63	>99.9	0.63	>99.9
PVDF 0.22 μm	50	1740	89.9	1980	88.6	<20	>94	4.8	94.1	0.89	>99.9	0.31	>99.9
	60	2430	85.9	2440	85.9	39	87.9	11.4	86	0.83	>99.9	0.41	>99.9
	70	4860	71.6	3812	77.9	46	85.7	12.6	84.5	1.17	>99.9	0.67	>99.9
	80	7890	53.9	4566	73.6	158	50.9	42.5	47.7	71.73	>99.9	1.17	>99.9
PVDF 0.45 μm	50	1960	88.6	2110	87.8	42	87	12	85.2	1.57	>99.9	0.69	>99.9
	60	2520	85.3	2651	84.7	54	83.2	13.5	83.4	19.62	>99.9	0.91	>99.9
	70	5110	70.2	3910	77.4	216	32.9	57.6	29.2	26.07	>99.9	0.93	>99.9
	80	9230	46.1	4690	72.8	233	27.6	58.6	27.9	68.24	>99.9	1.42	>99.9

Kesieme et al. (2014) reported a successful use of PTFE membrane in DCMD to extract acid and water from an actual leach solution. They achieved an overall 80% water recovery, 99.9% sulfate and salt rejection (Kesieme et al. 2014). A similar study with MD succeeded in removing 90.87% of sulfate, 99.58% chloride, 87.99% COD, and 95.51% conductivity (Zarasvand Asadi et al. 2012), and over 96% iron, aluminum, TOC, conductivity, COD, and sulfate (Noor et al. 2020). The contaminants removal trend in the present study exhibited in Table 4 is in conformity with the findings reported in previous studies. Furthermore, according to Xu et al. (2016), the removal efficiency of contaminants in the MD process demonstrated a relatively stable trend for all membrane pore sizes not exceeding 0.45 μm. However, a significant decline in removal efficiency was observed for pore sizes beyond 0.45 μm (Xu et al. 2016). The present study found that the PVDF membrane with a pore size of 0.22 μm exhibited higher contaminant removal compared to PVDF 0.45 μm. In contrast, no significant difference was observed between PTFE 0.22-μm and 0.45-μm membranes, suggesting a more advantageous application of the PTFE 0.45-μm membrane for the treatment of anodic oxidation wastewaters.

**Fig. 2:**
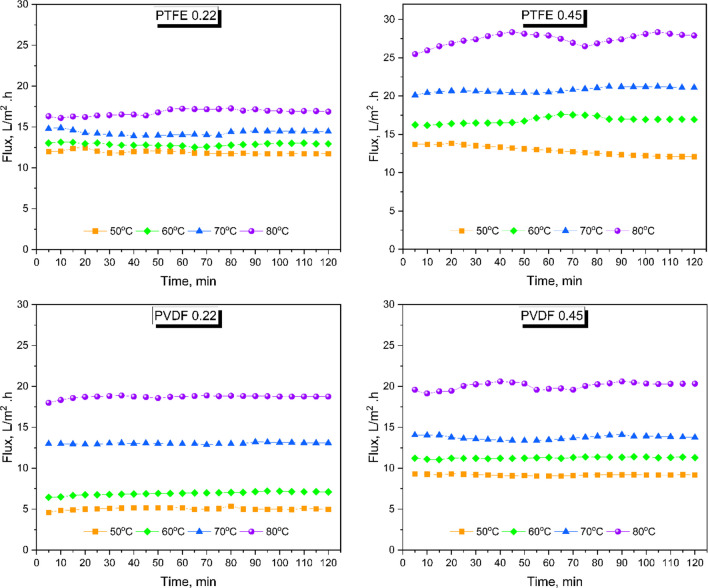
Flux of PTFE and PVDF Membranes at various feed temperature

An important advantage of membranes with larger pore sizes, as opposed to those with smaller pores, is the higher mass transfer coefficient, leading to a substantial increase in membrane flux. The results of membrane fluxes depicted in Fig. 2 indicate that the flux values increased in proportion to the feed water temperature and membrane pore size. The flux remained relatively stable over the 2-h operation period for all membranes tested at each temperature. In most operational conditions, PTFE membranes consistently achieved higher flux compared to PVDF membranes, regardless of their pore size. Previous studies have reported that PTFE membranes yield higher flux than PVDF under identical operating conditions. The membrane support layer for PTFE and PVDF not only influences the flux but also affects the energy efficiency of the process (Zhang et al. 2010). Li et al. (2018) reported that the PTFE membrane consistently exhibits improved flux and rejection capabilities for certain distinctive contaminants compared to PVDF membrane mainly due to their superior hydrophobic properties and lower wettability (Li et al. 2018).

At feed temperatures 50, 60, 70, and 80 °C, the average flux for the 0.22 μm PTFE membrane were 11.90, 12.86, 14.29, and 16.79 L/m^2^ h respectively. Comparatively, the 0.45 μm PTFE membrane achieved substantially higher flux, with average flux values of 12.90, 17.63, 20.79, and 27.44 L/m^2^ h respectively, at the same temperatures. The increase of both feed temperature and the recirculation flow rate induces a decrease in membrane resistance considered as the the primary contributor to the overall mass transfer resistance (Foureaux et al. 2021). The rise in flux with increasing feed temperatures had a mild impact on contaminants’ removal efficiency in PTFE membranes, while in PVDF membranes, it significantly influenced the removal performance. The 0.22-μm PVDF membrane achieved mean flux values of 5.03, 6.93, 13.04, and 18.72 L/m^2^ h at the same feed water temperatures. The PVDF membrane had higher flux with a larger pore diameter, similar to the PTFE membrane. The 0.45-μm PVDF membrane had an average flux of 9.17, 11.27, 13.75, and 20.08 L/m^2^ h at the same temperatures. The size of the PTFE membrane’s pores played a vital role in determining both the transmembrane flux and effluent quality, as illustrated in Fig. 2 and Table 4. The study findings suggest that at low feed water temperature, an increase in membrane pore size from 0.22 to 0.45 μm induces a comparable effect on flux as raising the feed temperature from 50 °C to 60 °C. However, at higher temperatures, the influence of increasing the pore size becomes more pronounced than elevating the feed temperature from 70 to 80°C. Moreover, the relationship between feed temperature and flux varied depending on the membrane pore size, with an increase in feed temperature causing more water molecules to evaporate and, consequently, an increase in flux, particularly in membranes with 0.45-μm pore diameters. Although feed temperature was an essential factor affecting the flux, the pore size of the membrane also played a crucial role. In particular, the larger pore diameters in the 0.45-μm membranes facilitated more water vapor transmission at high feed solution temperatures, thereby leading to a more significant influence of feed temperature on the flux. Similar flux behaviors were observed in previous studies. Noor et al. achieved a flux of 14.8 L/m^2^·h using 0.20-μm PTFE membranes operated at a feed temperature of 70°C (Noor et al. 2020). Zhang et al. reported that, under the same conditions, PTFE membranes with a higher contact angle and larger pore diameter achieved a greater flux compared to other membranes (Zhang et al. 2010). In a comparative study conducted by Kim et al., the results showed that both the contact angle and the flux of the PTFE membrane were larger than those of the PVDF membrane (Kim et al. 2021).

### Main properties of PTFE and PVDF membranes influencing their performance

While membrane pore size is considered an important property, the different membranes used exhibited variations in pore distribution, membrane thickness, membrane porosity, tortuosity, and LEP, all of which contributed to the performance of the membranes and their resistance to wetting during the treatment of anodic oxidation wastewater. As a fact in MD processes, larger membrane pore sizes are required for higher flux, but smaller pore sizes are necessary to prevent liquid penetration and membrane wetting. Therefore, the optimal pore size should be determined based on the feedwater and operating conditions. Additionally, pores on the membrane surface are generally not uniformly distributed, which can affect membrane flux. Conversely, as membrane thickness increases, mass transfer resistance also increases, leading to a reduction in flow. Moreira et al. (2023) investigated the treatment of wastewater generated by gold mining operations with MD using PP, PTFE and PVDF membranes and reported that the PP membrane exhibited the highest flux owing to its reduced thickness in comparison to the other membranes. On the other hand, the PTFE membrane achieved an exceptional separation efficiency, over 99.2% of pollutants removal (Moreira et al. 2023). A thinner membrane promotes higher membrane permeability, while a thicker membrane reduces heat loss and might perform better in contaminants rejection. The right thickness will achieve good contaminant rejection and still maintain a reasonably high flux. PTFE 0.45-μm membrane in the present study depicted such features. Membranes with high porosity offer more surface area for evaporation, resulting in increased transmembrane flux. However, high tortuosity can lead to reduced flux (Drioli et al. 2015; Essalhi and Khayet 2015). All such various properties should be taken into account while choosing the right membrane for a given water treatment in an MD process (Essalhi and Khayet 2015).

Comparing both PVDF and PTFE membranes used in the present study, the lower thickness of PVDF membranes provided an advantage in terms of membrane flux. However, such membranes may be less selective, resulting in lower effluent quality of treated anodic oxidation wastewater compared to PTFE membranes. Additionally, the lower thickness of PVDF membranes renders them more susceptible to fouling and wetting. PTFE membranes have the advantage of high porosity and excellent laminating strength. Both 0.22- and 0.45 PTFE-μm membranes exhibit good uniformity and pore distribution on the membrane surface, along with a low friction coefficient. They possess uniform pore distribution and high pore volume, with membrane porosity ranging between 85 and 93% (Omnexus n.d.-a). (An et al. 2016) reported in a study that PTFE membranes exhibited greater porosity and hydrophobicity compared to the PVDF membranes leading to an increase in flux and served as a preventive measure against long-term fouling. These attributes along with polyethylene terephthalate (PET) used as a support material, contribute to a higher LEP of PTFE membranes (> 5 bars) compared to PVDF membranes (<4 bars). PTFE membranes are, therefore, considered best suited for controlling membrane wetting, exhibiting consistent behavior, and achieving an overall better removal of contaminants (Xie et al. 2022), and higher membrane flux with larger pore-size membranes.

### Membrane distillation performance with 24-h experiment

The PVDF membranes used in the study exhibited lower performance in contaminant removal, failing to achieve satisfactory anodic oxidation wastewater treatment. On the other hand, all PTFE membranes achieved satisfactory removal of pollutants for anodic oxidation wastewater discharge. The PTFE 0.22-μm membrane exhibited the highest contaminant removal efficiency, while the PTFE 0.45-μm membrane was found to be the most suitable for achieving a high average flux while still meeting the target for contaminants removal in wastewater discharge. To assess the flux behavior over time, the MD process was carried out for 24 h at a feed solution temperature of 60°C using the PTFE 0.45-μm membrane. Over the 24-h operation, filtrate samples were continuously collected every 3 h to monitor flux dynamics and the membrane’s performance in contaminant removal. This resulted in a total of eight samples. The membrane flux behavior is presented in Table 5.

**Table 5:** Membrane flux values for 24-h membrane distillation treatment at 60°C

Membrane type and feed temperature	Time (h)	Average Flux (L/m^2^h)
PTFE 0,45 μm - 60°C	0-3	17.65
	3-6	17.60
	6-9	17.49
	9-12	17.20
	12-15	17.09
	15-18	16.91
	18-21	16.90
	21-24	16.89

The results in Table 5 reveal a continuous decline of the flux until the 15th hour, followed by a slower rate of decrease. Throughout the process, contaminants accumulate in the tank holding the feed due to the recycling of the concentrate flow. However, despite the gradual increase in pollutant concentration in the tank, the flux value remained relatively stable and was not significantly affected. Over the course of more than 40 h of operation, (S. Zhang et al. 2014) found only a negligible drop in flux during an MD process. Kim et al. reported that both the feed solution pH and operation time significantly affect the MD flux. However, they found that the flux reached a stable point between 61 and 65 h of operation (S. Kim et al. 2016). Similar results were reported by Peng et al. when they conducted an MD process at 70°C for an extended period of time (Peng et al. 2005). In the present study, a slight decline of the flux was observed during the initial hours of operation, with flux stability achieved after the 15th hour. The hydrophobic PTFE membrane has the ability to repel the feed solution from its surface, allowing only water vapor to come into contact and cross the membrane. As a result, membrane wetting and fouling are minimized. Furthermore, the membrane’s structure endows it with resistance to variations in feed solution temperature and feed water pressure, ensuring the membrane maintains its integrity throughout the operation. Similar results were reported by (Foureaux et al. 2021) with PTFE and PVDF membranes operated for 240 days in the treatment of wastewater from the mining industry. Both membranes exhibited consistent rejection efficiency and distillate flux even after being exposed to wastewater for over 8 months. Moreover, there were no notable alterations detected in the membranes’ morphology or structure.

Table 6 indicates that there were minor fluctuations in the rejection efficiency during the 24-h MD operation at 60 ^o^C. The removal efficiencies for iron, aluminum, COD, sulfate, TOC, and conductivity remained consistently high, above 99.9%, 99.9%, 94%, 99.7%, 89.1%, and, 99.4%, respectively. In another study conducted by Ali et al., the effectiveness of membrane distillation and membrane crystallization for treating wastewater from the anodizing industry was investigated. The MD/MCr unit was able to convert approximately 90% of the wastewater into useful products such as fresh water and salt crystals (Ali et al. 2019). The PTFE membrane was able to maintain its integrity over the 24-hoperation, membrane wetting did not occur, and all contaminants in the feed solution were continuously removed with a stable and constant rejection efficiency.

**Table 6:** Fluctuations in rejection efficiency of 24-h PTFE 0,45-μm MD wastewater treatment at 60°C

Time	Conductivity		Sulfate		COD		TOC		Aluminum		Iron	
H	μS/cm	%	mg/L	%	mg/L	%	mg/L	%	μg/L	%	μg/L	%
0–3	101.6	99.4	51.5	99.7	<20	>94	8.9	89.1	0.48	>99.9	0.45	>99.9
3–6	101.6	99.4	51.5	99.7	<20	>94	8.9	89.1	0.48	>99.9	0.45	>99.9
6–9	101.6	99.4	51.5	99.7	<20	>94	8.9	89.1	0.49	>99.9	0.45	>99.9
9–12	101.6	99.4	51.5	99.7	<20	>94	8.9	89.1	0.47	>99.9	0.45	>99.9
12–15	101.7	99.4	51.5	99.7	<20	>94	8.9	89.1	0.48	>99.9	0.46	>99.9
15–18	101.7	99.4	51.5	99.7	<20	>94	8.9	89.1	0.49	>99.9	0.46	>99.9
18–21	101.6	99.4	51.5	99.7	<20	>94	8.9	89.1	0.49	>99.9	0.46	>99.9
21–24	101.6	99.4	51.6	99.7	<20	>94	9.1	89.1	0.49	>99.9	0.46	>99.9

### Structure and surface morphology’s influence on membrane performance

PTFE is a fluoropolymer with a chemical structure composed of polymerized CF_2_-CF_2_ units, forming a linear polymer. The fluorine atoms create a uniform and continuous sheath around the carbon-carbon bonds, providing excellent chemical resistance and stability to the molecule. This arrangement also grants the synthesized PTFE structure exceptional chemical resistance, heat tolerance, and good electrical insulating properties in hot and wet environments. The non-stick and slippery surfaces of PTFE membranes confer them with anti-adhesion properties, allowing them to resist fouling during MD processes (Omnexus n.d.-a). On the other hand, PVDF is a semi-crystalline thermoplastic fluoropolymer, consisting of a chain of alternating CH_2_ and CF_2_ groups. Its synthesis occurs through the polymerization of the PVDF monomer, 1,1-difluoroethylene (CH_2_=CF_2_). The polarity of CH_2_ and CF_2_ groups leads to the insolubility and electrical properties of PVDF membranes. From a structural point of view, fluorinated polymers of PVDF membranes show chemical inertness to a narrower range of chemicals compared to PTFE (Omnexus n.d.-b).

**Fig. 3:**
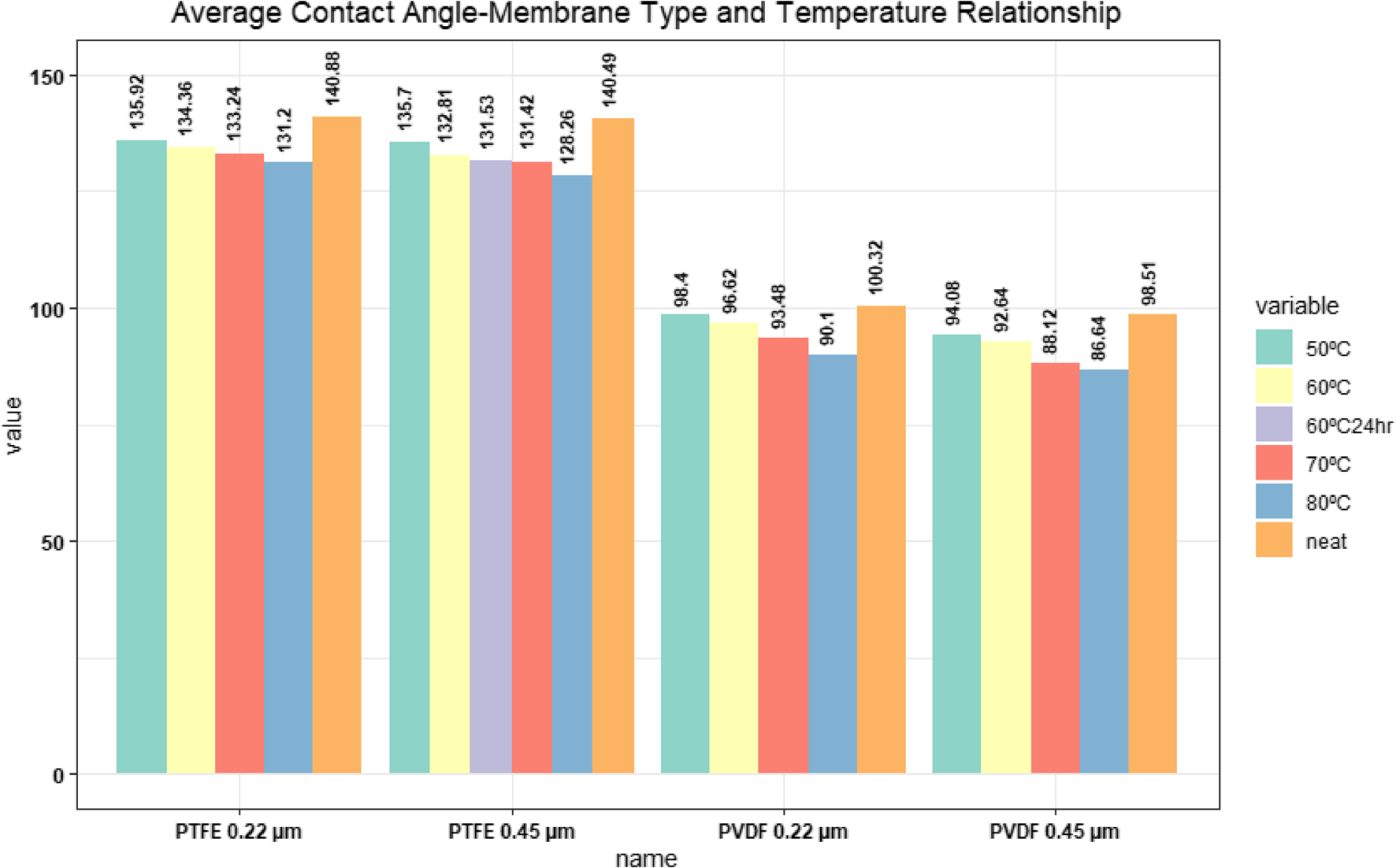
Average contact angle-membrane type and temperature relationship

To gain a deeper understanding of membrane fouling and wetting in membrane processes, various characterization techniques are required, such as contact angle measurements, SEM, and FTIR. These techniques were employed to investigate the surface chemistry of both clean and neat membranes, with the aim of shedding light on the mechanisms underlying membrane fouling and wetting (Fortunato et al. 2016; Wu and Fane 2012). The surface morphology of the membrane plays a crucial role in influencing its performance (Childress and Brant 2000). The results revealed that an increase in feed water temperature led to a slight decrease in the contact angle of the PTFE membranes (as shown in Fig. 3), suggesting a minor loss in membrane hydrophobicity. Membranes that are hydrophobic in nature typically have contact angles that exceed 90°, with contact angle values increasing with greater hydrophobicity. When pore size and temperature increase, water can interact more easily with the membrane pores, leading to an increase in flux and a decrease in contact angle. The results of the short-term experiments conducted in the study revealed a surprising decrease in membrane hydrophobicity upon raising the feed water temperature. Consequently, the study highlights that membrane pore size, in addition to temperature, is a crucial factor that influences membrane performance. Larger pore diameters facilitate greater water vapor transmission, particularly evident in membranes with pore sizes of 0.45 μm, thereby underscoring the importance of pore size in determining the effectiveness of the membrane. Overall, these findings highlight the need for comprehensive characterization techniques to gain a better understanding of the behavior of membrane systems.

The findings reported in Fig. 3 show that the anodizing wastewater influences the membrane structure. As the feed water temperature increases, more steam is generated, and the impact of this temperature increase on the contact angles of the membranes becomes evident. Specifically, the contact angle of the membranes is a useful indicator of the changes in their hydrophobicity due to increasing feed water temperature. This effect is not only related to the characterization and temperature of the water, but also to the membrane structure. When PTFE membranes are examined, the initial high hydrophobicity value is still maintained at a very high level even though it decreases with increasing feed water temperature. This situation can also be explained by the fact that the permeate quality is affected by the increasing feed water temperature to a very small extent. The PVDF membranes had an initial contact angle of around 90^o^, and the increase in feed water temperature resulted in the decrease of the contact angle to 90^o^ and below, significantly reducing the permeate quality. It is evident that the increase in the pore size of the membranes renders them more susceptible to wetting, as depicted in Fig. 3. Similar results about membrane wetting and fouling were reported by (Zoungrana et al. 2016) and (Khaing et al. 2010).

**Fig. 4:**
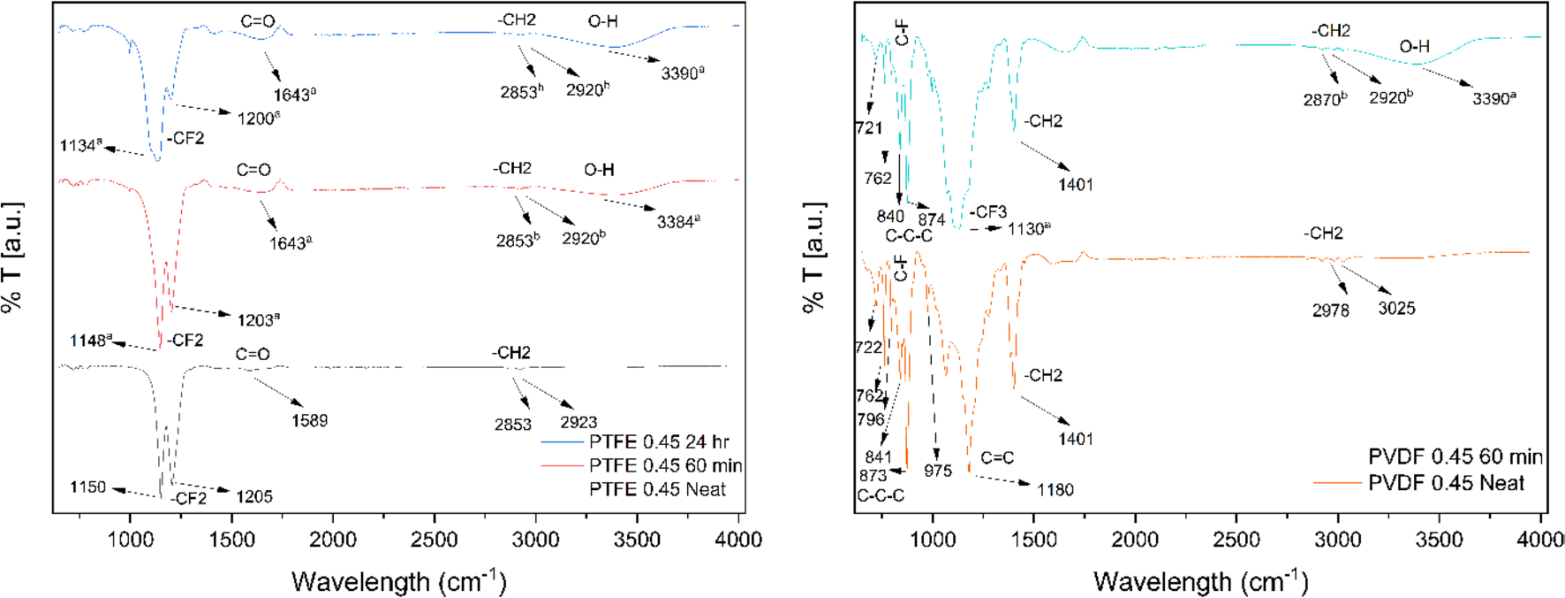
FTIR spectra of the neat and dirty PTFE and PVDF membranes: **a** stretching, **b** anti-symmetric vibration

FTIR spectroscopy is a widely used analytical technique across fundamental research, medicine, and engineering, particularly for identifying bonds within molecular structures. Analysis of FTIR spectra is a useful tool for understanding how membrane structure and major functional groups respond to various conditions (Alpatova et al. 2015; Hu et al. 2016; Vargo et al. 1991; Wang et al. 2015; Xue et al. 2015; Zhao et al. 2010). Figure 4 presents the primary peaks identified during the analysis of both used and unused membranes. The results indicate that the clean PTFE membrane exhibits two distinct peaks at 1205 cm^−1^ and 1150 cm^−1^. These peaks are associated with -CF_2_ and -CF_3_ group stretching vibrations (Wang et al. 2014; Wang et al. 2015). PTFE membranes showed no significant difference in peak patterns between wastewater treated for 2 h and 24 h at 60°C. However, peak stretching vibrations measured after 24 h were found to be less than those measured after 2 h. The used membranes showed a new peak development at 3390 cm^-1^, which may be due to intermolecular O-H stretching of carboxyl, hydroxyl, and phenol groups, while the peaks detected in the 2920–2853 cm^−1^ range are -CH_2_ group (Hu et al. 2016; Xue et al. 2015). The 750–720 cm^−1^ peaks observed in used membranes are due to the -CH2 group’s shaking vibrations; the 1643 cm^−1^ peak, on the other hand, may be due to the C=C stretching vibration. Compared to other used membranes, the peak of 1130 cm^−1^ occurring on the membranes after 24 h of operation may be related to the reduction in stretching vibration of the –CF_3_ group or to sulfate ions (Coates 2006).

The peaks displayed by the clean PVDF membrane at 3025 and 2978 cm^−1^ are due to the vibrations of the asymmetric and symmetric CH_2_ group. In the PVDF membrane, the peak at 1401 cm^-1^ is associated with the ripple vibration of the CH_2_ group, while the peak at 1180 cm^-1^ is associated with the C-C bonds (Bai et al. 2012). Furthermore, the peaks in the 981–750 cm^-1^ range correspond to the α and β phases of the PVDF membranes, while the peaks in the 878–839 cm-1 interval are associated with the asymmetric C-C-C and C-F stretch vibrations (Alpatova et al. 2015; Wang et al. 2014). After treating wastewater at 60 °C, a new peak formed at 3390 cm^-1^ on the PVDF membrane, which was observed in the used PTFE membranes and may be caused by intermolecular O-H stretching of carboxyl, hydroxyl, and phenol groups. Another possible explanation for the peaks between 2920 and 2870 cm^-1^ is that the -CH_2_ group is being stretched next to the -OH group (Hu et al. 2016; Xue et al. 2015). Another peak detected on the used membrane has a wavelength of 1130 cm^-1^ and may relate to sulfate ions or a decrease in the stretching vibration of the -CF_3_ group (Coates 2006).

**Fig. 5:**
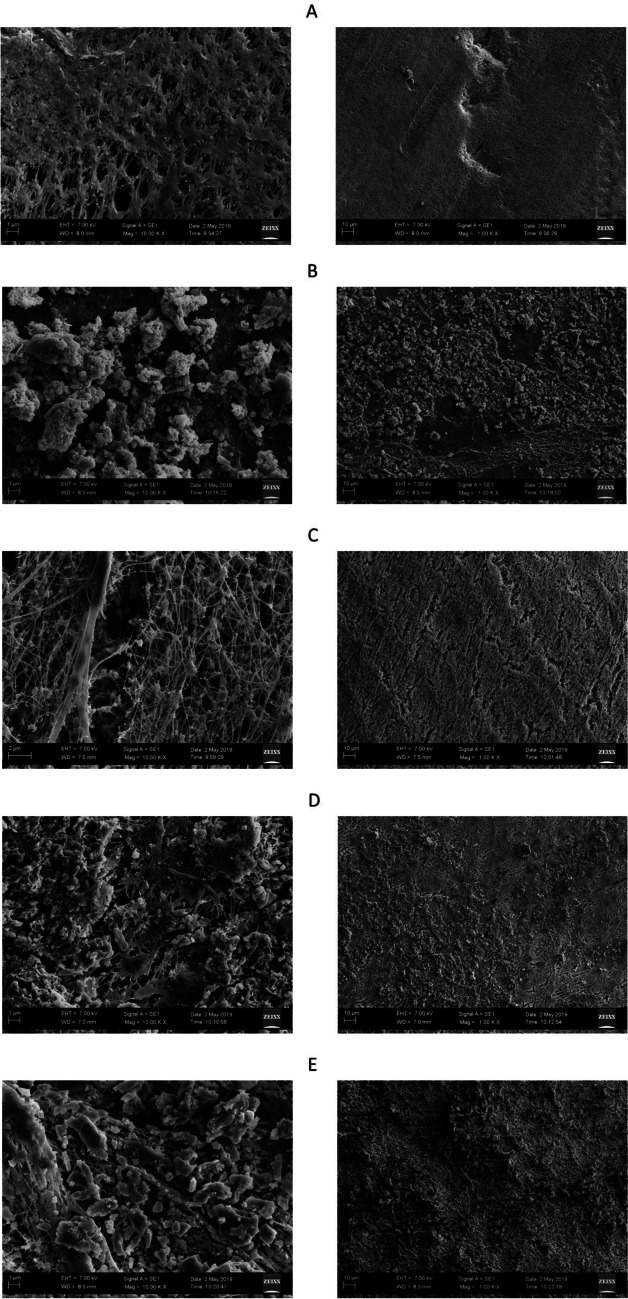
SEM: **A** Neat 0.4-μm PVDF membrane 1000 (right) and 10000 (left) magnification, **B** 60° C feed temperature after 2-hMD process 0.45-μm PVDF membrane 1000 (right) and 10000 (left) magnification, **C** neat 0.45-μm PTFE membrane 1000 (right) and 10000 (left) magnification, **D** 60° C feed temperature after 2 h-MD process 0.45-μm PTFE membrane 1000 (right) and 10000 (left) magnification, **E** 60° C feed temperature after 24-h MD process 0.45-μm PTFE membrane 1000 (right) and 10000 (left) magnification

SEM analysis provides valuable insights into both the topography and elemental composition of a material. Figure 5 showcases the topographic features of the membranes. Notably, micrographs of the clean membranes revealed a more uniform surface distribution on the PTFE membrane compared to the PVDF membrane. SEM analysis further revealed the deposition of organic materials on the membrane surfaces. PVDF membranes exhibited a higher degree of organic material deposition compared to PTFE membranes. This observation may explain the lower permeate flux and contaminant rejection performance observed in PVDF membranes. The micrographs of PTFE membranes revealed a superficial deposition of particles, potentially due to the presence of open holes on the membrane surface. The progressive accumulation of particles on the membrane surface might explain the decline of the permeate flux with time. The FTIR data, namely the O-H tensions at the 3390 cm^-1^ peak and the probable sulfate ions at the 1130 cm^-1^ peak, may have their origins in the accumulations shown in the membrane micrographs. (Xie et al. 2022) achieved similar findings of PTFE membranes exhibiting better fouling resistance compared to PVDF membranes. They argue that the surface scaling remained significant for the PVDF membrane due to its relatively low hydrophobicity, which failed to effectively impede the occurrence of heterogeneous nucleation on the membrane. As mentioned in previous sections, membrane pore sizes over 0.45 μm can induce lower performance, a point supported by their study, experiencing scaling with larger pores. They recommended a decrease in pore size and an increase in membrane hydrophobicity as a solution to enhance anti-scaling characteristics. This approach effectively mitigates the impact of both homogeneous and heterogeneous nucleation on the formation of gypsum scale.

## Conclusion

Wastewater from the anodized plating industries poses serious threats to aquatic ecosystems and must be treated before being discharged. Third-generation progressive treatment approaches, such as MD, are required to treat wastewaters with such high concentrations of contaminants since conventional treatment methods fail to do so. The performance of PTFE and PVDF membranes in removing contaminants from anodic oxidation wastewaters are mostly correlated to their structure, controlling both contaminants rejection efficiency and transmembrane flux.

Both 0.22- and 0.45-μm PTFE membranes effectively achieved the targeted water quality standards for anodic oxidation wastewater treatment across all tested feed temperatures, demonstrating excellent removal efficiencies exceeding 99% for conductivity, sulfate, aluminum, and iron and over 85.7% for organic matter removal. In contrast, PVDF membranes failed to meet the discharge requirements, especially at higher feed temperatures. A clear correlation between the pore size of the membranes, feed temperature, and removal efficiency was observed for both membrane materials. Smaller pore size membranes achieved better contaminant removal while maintaining lower fluxes throughout the MD treatment process.

While minor wetting was observed with PTFE membranes, fouling and wetting of PVDF membranes led to a decline in flux and significantly affected the permeate quality over time. The structure and morphology of PTFE membranes endowed them with slippery and anti-adhesion properties stronger than PVDF membranes, preventing fouling and wetting and maintaining their performance during MD operations for anodic oxidation wastewater treatment.

## Data Availability

All data generated or analyzed during this study are included in this published article.

## Abbreviations/Symbols

PTFE: polytetrafluoroethylene
PVDF: polyvinylidene fluoride
MD: membrane distillation
TOC: total organic carbon
COD: chemical oxygen demand
EC: European Council
pH: potential of hydrogen
MCr: membrane crystallization
PP: polypropylene
DCMD: direct contact membrane distillation
et al.: et alia
USA: United States Of America
MN: Minnesota
LEP: liquid entry pressure
SM: standard methods
APHA: American Public Health Association
Eq: equation
SEM: scanning electron microscopy
FTIR: Fourier transform infrared spectroscopy
n.d.: no date
PET: polyethylene terephthalate
NaOH: sodium hydroxide
Ca(OH)_2_: calcium hydroxide
•OH: hydroxyl radical
%: percentage
/: per
μS: microSiemens
cm: centimeter
m: meter
°: degree
mg: milligram
L: liter
m^2^: square meter
°C: Celsius degree
μm: micrometer
min: minute
cm^2^: square centimeter
mL: milliliter
R: membrane rejection
J: membrane permeate flux
C_f_: feed solution contaminant concentration
C_p_: permeate solution contaminant concentration
Q_P_: permeate flow rate
A: membrane surface area
CF_2_: difluorocarbene
CH_2_: methylene
C: carbon
H: hydrogen
